# Just-in-Time Correntropy Soft Sensor with Noisy Data for Industrial Silicon Content Prediction

**DOI:** 10.3390/s17081830

**Published:** 2017-08-08

**Authors:** Kun Chen, Yu Liang, Zengliang Gao, Yi Liu

**Affiliations:** 1 Department of Electrical and Information Engineering, Shaoxing University, Shaoxing 312000, China; kchen@usx.edu.cn; 2 Institute of Process Equipment and Control Engineering, Zhejiang University of Technology, Hangzhou 310014, China; yuliang@zjut.edu.cn (Y.L.); zlgao@zjut.edu.cn (Z.G.)

**Keywords:** soft sensor, industrial blast furnace, silicon content, local learning, support vector regression, outlier detection

## Abstract

Development of accurate data-driven quality prediction models for industrial blast furnaces encounters several challenges mainly because the collected data are nonlinear, non-Gaussian, and uneven distributed. A just-in-time correntropy-based local soft sensing approach is presented to predict the silicon content in this work. Without cumbersome efforts for outlier detection, a correntropy support vector regression (CSVR) modeling framework is proposed to deal with the soft sensor development and outlier detection simultaneously. Moreover, with a continuous updating database and a clustering strategy, a just-in-time CSVR (JCSVR) method is developed. Consequently, more accurate prediction and efficient implementations of JCSVR can be achieved. Better prediction performance of JCSVR is validated on the online silicon content prediction, compared with traditional soft sensors.

## 1. Introduction

The silicon content is an important index of the thermal state in industrial blast furnaces. It must be controlled appropriately to facilitate stable production. To achieve this goal, the accurate state of silicon content in hot metal should be known online. Previously, there was extensive research on the kinetic and thermodynamic behaviors occurring in the blast furnace iron-making process [1,2,3,4,5,6]. Nevertheless, an accurate mechanism model suitable for industrial applications is still not available. Nowadays, process data can be easily measured in industrial blast furnaces. To explore and utilize useful information hidden in the data, several empirical soft-sensing approaches were applied to online predict the silicon content. Existing popular data-driven methods include artificial neural networks [7,8,9,10,11,12,13,14], multivariate regression [14,15], time series analysis [16,17,18,19], fuzzy systems [20], subspace identification [21], support vector regression (SVR) and least squares SVR (LSSVR) [22,23,24,25], and multi-scale and multiple models [26,27,28,29,30]. These data-driven empirical models for short-term silicon content prediction can be constructed in a quick way [31,32,33].

Actually, the development of a good soft sensor model is easy. This is mainly because the modeling data are noisy and often contain unwanted outliers. They may come from instrument degradation, transmission problems, etc. Generally, a good soft sensor model is dependent on the high quality of modeling data. Different kinds of noise should be considered when training artificial neural networks and other data-driven models [34,35,36]. Without enough attention, a soft sensor model trained with outliers and inappropriate data may tend to be over-fitting and, thus, lead to unreliable prediction. For practical use, a reliable prediction model should be constructed by reducing the negative effect of outliers. Generally, obvious outliers can be deleted by most of traditional outlier detection methods [37,38,39,40]. However, it is not easy to detect those inconspicuous outliers mainly because they may be masked by their adjacent data. In our opinion, the soft sensor development and outlier detection should be integrated into a unified framework rather than be separated into two tasks. 

For industrial blast furnace iron-making processes, only using a global/fixed model is not possible to describe the complex characteristics. Additionally, it is difficult to update the global models quickly when the process dynamics are changing [41]. Nurkkala et al. [30] proposed multiple autoregressive vector models to describe complex systems. To construct the local models automatically, several just-in-time learning methods were utilized for nonlinear process modeling problems [42,43,44]. Different from most traditional soft sensors, just-in-time-based models are built in a lazy learning manner when the query sample is required to be predicted. Consequently, the advantage is that the prediction for the query sample can be optimized locally, which might increase the prediction performance. For the silicon content prediction, Liu and Gao [25] utilized the just-in-time LSSVR (JLSSVR) modeling method to better describe process nonlinearity directly. Unfortunately, data samples utilized for construction of a JLSSVR model are assigned with the same weight regardless of their different effects. In such a situation, the negative effect of outliers may not be removed.

A novel online local model is developed for reliable prediction of the industrial silicon content. To handle the noisy data with non-Gaussian and uneven distributions, a just-in-time correntropy SVR (JCSVR) soft sensor is proposed. Compared with traditional soft sensors, the proposed JCSVR method is more reliable and practical in two ways. First, by reduction of the outliers’ negative effect, more accurate prediction of the silicon content can be obtained. Second, the reliability of the database can be improved gradually. These two properties make the JCSVR method better for long-term utilization.

The remainder of this work is structured thusly: The correntropy SVR (CSVR) soft sensing approach is formulated in Section 2. In Section 3, the clustering-based JCSVR local modeling method is proposed. Additionally, the database maintenance is implemented. In Section 4, the JCSVR method is applied to online silicon content prediction. Finally, a conclusion is drawn in Section 5.

## 2. CSVR-Based Soft Sensor Model

In this section, how to integrate the maximization correntropy criterion [45,46] and SVR into a CSVR-based unified framework is formulated. The CSVR model *f* is to fit a dataset {S}={X,Y}, where $\left\{X\right\}={\left\{x_{i}\right\}}_{i=1}^{N}$ and $\left\{Y\right\}={\left\{y_{i}\right\}}_{i=1}^{N}$ are *N* input and output data samples, respectively. The relationship is formulated as [45]:

(1) $$y_{i}=f\left(x_{i};\theta \right)+e_{i}=f\left(x_{i};w,b\right)+e_{i}=w^{T}\phi \left(x_{i}\right)+b+e_{i},\;i=1,\cdots ,N$$

where *f*(·) is the model; *e_i_* is the noise item of the *i*th sample; x*_i_* is an input vector composed of several online-measured variables. The model parameter vector and the bias are **w** and *b*, respectively, and $\theta ={\left[w^{T},b\right]}^{T}$. The CSVR model is solved using the optimization problem below [45]:

(2) $$\left\{ \begin{array}{l} \min\;J\left(w,b,\rho \right)=\frac{\gamma }{2}\sum_{i=1}^{N}\rho \left(e_{i}\right)e_{i}^{2}+\frac{1}{2}{\left\|w\right\|}^{2}\\ s.t.\;y_{i}-w^{T}\phi \left(x_{i}\right)-b-e_{i}=0,\;i=1,\cdots ,N \end{array}\right.$$

where *γ* (*γ* > 0) is the regularization parameter determining the trade-off between the approximation accuracy and the model’s complexity. Several approaches [45] are available for selection of the kernel width *σ* of the related items $\rho \left(e_{i}\right)=\frac{\exp\left(-\frac{e_{i}^{2}}{2\sigma^{2}}\right)}{\sigma^{3}\sqrt{2\pi }}$. Here, it is simply adopted as $\sigma =\frac{\max\left|e_{i}\right|}{2\sqrt{2}},\;i=1,\cdots ,N$ [46].

According to Liu and Chen [46], a two-step iterative algorithm is adopted to obtain the solution of above problem in Equation (2). Finally, a CSVR soft sensor model is established. For a test sample $x_{t}$, its prediction ${\hat{y}}_{t}$ is formulated below:

(3) $${\hat{y}}_{t}=f(w,\;b;x_{t})=\sum_{i=1}^{N}\alpha_{i}\langle \phi \left(x_{i}\right),\phi \left(x_{t}\right)\rangle +b=\alpha^{T}k_{t}+b$$

where $k_{t}={\left[k_{t1},\cdots ,k_{tN}\right]}^{T}\in R^{N\times 1},k_{ti}=\langle \phi \left(x_{t}\right),\phi \left(x_{i}\right)\rangle ,\;\forall i=1,\cdots ,N\;$ is a kernel column.

For an established CSVR model, the corresponding weight of a training sample $x_{i}$ is $\rho \left(e_{i}\right)=\frac{\exp\left(-\frac{e_{i}^{2}}{2\sigma^{2}}\right)}{\sigma^{3}\sqrt{2\pi }}$. Using the weights, the uncertainty of the training data can be quantified. Generally, the outliers are only a small portion of all data, and they can be automatically assigned with relatively smaller weights [46]. Consequently, those candidate outliers can be identified using a simple criterion in Equation (4):

(4) $$\rho \left(e_{i}\right)<\bar{\rho }$$

where $\bar{\rho }$ is a cutoff value and it can be chosen as a small one less than 1 after simply normalizing all the weights $\rho \left(e_{i}\right),i=1,\cdots ,N$ into the range of [0, 1].

In summary, the candidate outliers can be detected simultaneously using the weights of an established CSVR model. Interestingly, although the outliers are temporarily not removed out, they cannot degrade the prediction performance of CSVR due to their relatively small weights [45,46]. At a glance, the CSVR method is similar with some weighted SVR methods, e.g., in [47,48]. However, most weighted SVR methods are heuristic [47,48]. For complex industrial data, it is difficult to design suitable weighted strategies. Unlike those heuristic schemes, a reliable CSVR model can be constructed more directly for noisy data.

## 3. Correntropy-Based Local Modeling and Prediction Method

### 3.1. JCSVR-Based Local Model

In this section, the JCSVR modeling method for online prediction of a query sample **x***_q_* is described. First, search similar samples in the database **S** as a similar set $S_{q}$ using some defined similarity criteria. Second, establish a JCSVR model *f*_JCSVR_(**x***_q_*) with $S_{q}$. Third, obtain ${\hat{y}}_{q}$ for **x***_q_* online. With the same implementations, a new JCSVR model can be constructed for another query sample.

As a common similarity, the Euclidean-distance-based similarity index (SI) is defined below [42]:

(5) $$SI_{qi}=\exp\left(-d_{qi}\right)=\exp\left(-\left\|x_{i}-x_{q}\right\|\right),i=1,\cdots ,N$$

where *d_qi_* denotes the similarity between **x***_q_* and **x***_i_* in the historical set. Obviously, $0\leq SI_{qi}\leq 1$. When $SI_{qi}$ approaches to 1, **x***_q_* and **x***_i_* are almost the same. Other similarity criteria (e.g., correlation-based similarity) [41,43,44] can also be utilized to search similar samples.

To select a suitable dataset $S_{q}$ with $n_{q}$ similar samples, the $n_{\max}$ most similar samples can be ranked using the SI criterion in Equation (5). Correspondingly, a cumulative similarity factor (CSF) $CSF_{qn}$ is defined below [44]:

(6) $$CSF_{qn}=\frac{\sum_{i=1}^{n_{q}}SI_{qi}}{\sum_{i=1}^{n_{\max}}SI_{qi}},\;n_{q}\leq n_{\max}$$

where $CSF_{qn}$ denotes the cumulative similarity of $n_{q}$ most similar samples of all $n_{\max}$ samples. The CSF index can determine the most similar samples simply. For example, $CSF_{qn}=0.85$ means 85% of the similar samples are chosen [44]. Using the similarity criterion, a similar dataset $S_{q}$ is utilized to construct the JCSVR model.

### 3.2. Implementations of the Proposed Method

In this section, the JCSVR-based online modeling method is enhanced for a relative long-term utilization. Generally, there are some outliers in the initial training dataset. In the offline modeling stage, the CSVR method is first applied to the initial training dataset. After this preprocessing step, some outliers can be identified. Additionally, to make the JCSVR method more efficient in computation, the training data are clustered into several groups. This can divide the whole dataset into several subsets. The data in each subset show similar characteristics. Consequently, for online prediction of a query sample, only its similar data are searched. This can improve the computation efficiency. The step-by-step procedures of the JCSVR-based modeling method for online silicon content prediction are summarized below:

**Step** **1.**  Collect the process input and output data, i.e.,{S}={X,Y}, for training of the CSVR model.
**Step** **2.**  Train a CSVR model using the common cross-validation training strategy [46]. The weights $\rho \left(e_{i}\right),\;i=1,\cdots ,N$ can be obtained simultaneously. Then normalize all the weights $\rho \left(e_{i}\right),\;i=1,\cdots ,N$ into the range of [0, 1]. Using Equation (4) to identify the outliers and assign them into a outlier set $S_{outlier}$. The relative clean dataset can be denoted as $S_{normal}=\left\{X_{normal},Y_{normal}\right\}$.
**Step** **3.**  Applying a simple fuzzy c-means (FCM) clustering approach [49] to $S_{normal}$, the training samples are clustered into *l* sub-classes, denoted as $\left\{S_{normal,1},S_{normal,2},\cdots ,S_{normal,l}\right\}$. For $X_{normal}$, each sub-class has a center denoted as $\left\{c_{normal,1},c_{normal,2},\cdots ,c_{normal,l}\right\}$.
**Step** **4.**  For online prediction of a new input measurement $x_{q}$, judge which center of the sub-classes $\left\{c_{normal,1},c_{normal,2},\cdots ,c_{normal,l}\right\}$ is its nearest one. If $c_{normal,j}$ is the nearest to $x_{q}$, only search the similar set $S_{q}$ in $S_{normal,j}$ using the similarity criterion (Equations (5) and (6)). A JCSVR model for $x_{q}$ can be online constructed and the prediction ${\hat{y}}_{q}$ is obtained.
**Step** **5.**  If new training data $S_{new}=\left\{X_{new},Y_{new}\right\}$ are available, combine these data into S (i.e., $S=S_{new}\cup S$) and go to step 1. Otherwise, go to step 4 and repeat the same procedure for online prediction of another new input $x_{q+1}$.

**Figure 1:**
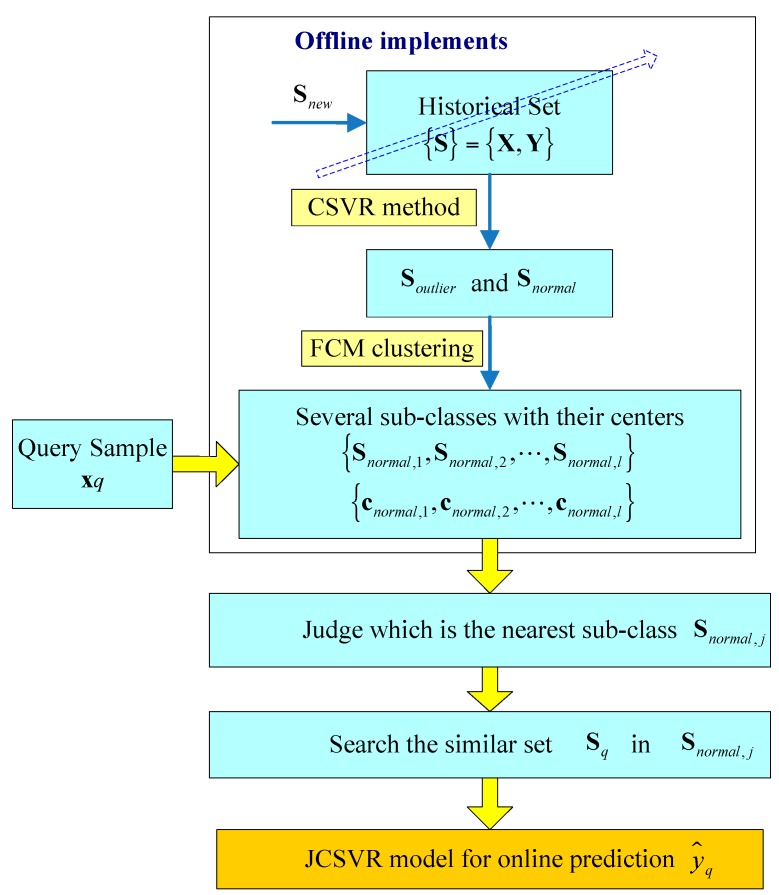
Main implemented steps of the proposed soft sensor modeling method integrating both of offline implements and online JCSVR method.

The main implemented steps of the JCSVR-based soft sensor modeling and prediction are summarized in Figure 1. For industrial data, candidate outliers are simply identified without considerable efforts. Additionally, step 2 and step 3 can be implemented offline. This can improve the computation efficiency for the online JCSVR modeling method. Consequently, the proposed JCSVR-based local method can provide a relative long-term utilization for the silicon content prediction.

## 4. Industrial Silicon Content Prediction

The presented JCSVR-based local modeling method is applied to online prediction of the silicon content in an industrial blast furnace in China. The input variables correlated with the silicon content include the blast temperature, the blast volume, the gas permeability, the top pressure, the top temperature, the ore/coke ratio, and the pulverized coal injection [21,22,24]. The sampling time of most of these input variables is 1 min.Additionally, the time difference between the silicon content and input variables is selected according to expert experience [31]. For example, the time difference between the silicon content and the top pressure is about 2 h. The silicon content is analyzed offline and infrequently. Consequently, the soft sensor is constructed using the online measured variables.

**Figure 2:**
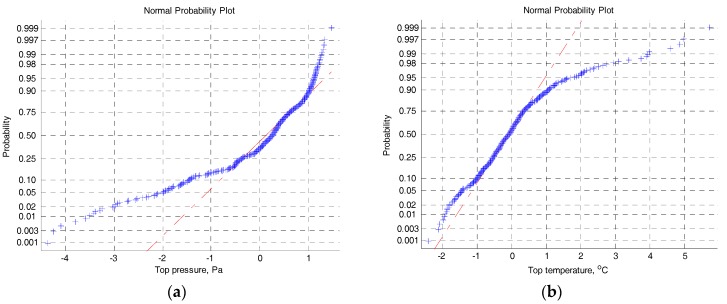
(**a**) The normal probability plot of the top pressure variable in the training set; and (**b**) the normal probability plot of the top temperature variable in the training set.

**Figure 3:**
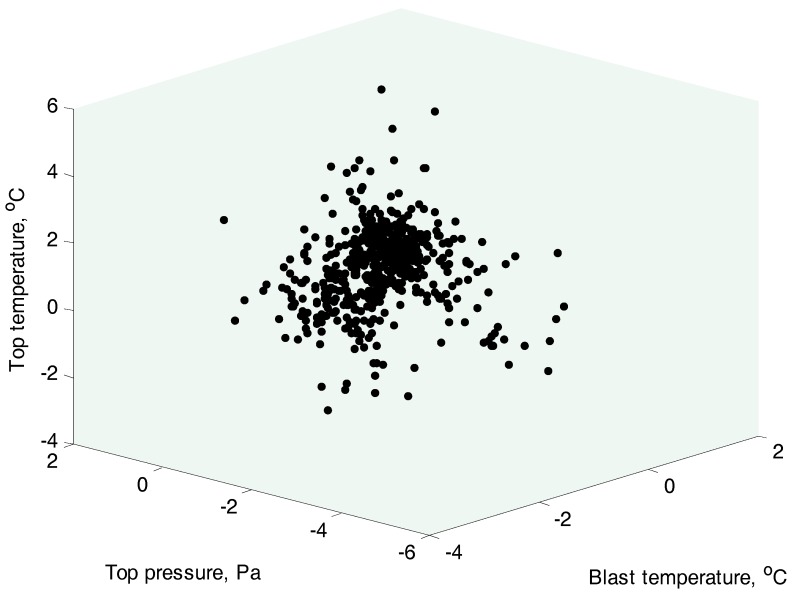
The spatial relationship of several process input variables in the training set.

After simply removing obvious outliers using the 3-sigma criterion, a set of 440 data samples is investigated. The historical set consists of 240 data. The rest 200 data points are for testing. It should be noted that the data are noisy and still contain some inconspicuous outliers. The normal probability of two input variables, including the top pressure and the top temperature, is shown in Figure 2a,b, respectively. The distribution results indicate that the process variables violate the Gaussian distribution denoting by the red lines in Figure 2a,b, respectively. The other process variables not plotted here are also non-Gaussian distribution. Additionally, as illustrated in Figure 3, several input variables exhibit the nonlinear relationship, and the data in different operating areas are distributed irregularly.

To show the advantage of JCSVR, it is compared with three SVR-based soft sensors, including JLSSVR [25], CSVR [46], and LSSVR [47]. To evaluate the prediction performance of different models, three indices of the root-mean-square error (RMSE), relative RMSE (simply noted as RE), and the hit rate (HR) [21,22,23,24,25,26,27,28,29,30,31] are utilized and defined below, respectively:

(7) $$\mathrm{RMSE}=\sqrt{\sum_{q=1}^{N_{\mathrm{tst}}}{\left(\frac{y_{q}-{\hat{y}}_{q}}{N_{\mathrm{tst}}}\right)}^{2}}$$

(8) $$\mathrm{RE}=\sqrt{\sum_{q=1}^{N_{\mathrm{tst}}}{\left(\frac{y_{q}-{\hat{y}}_{q}}{y_{q}}\right)}^{2}/N_{\mathrm{tst}}}$$

(9) $$\left\{ \begin{array}{l} \mathrm{HR}=\frac{\sum_{q=1}^{N_{\mathrm{tst}}}H_{q}}{N_{\mathrm{tst}}}\times 100\%\\ \mathrm{where}\;H_{q}=\left\{ \begin{array}{l} 1,\;\left|{\hat{y}}_{q}-y_{q}\right|\leq 0.1\\ 0,\;\mathrm{else} \end{array}\right. \end{array}\right.$$

where $y_{q}$ and ${\hat{y}}_{q}$ are the actual value and the predicted value, respectively, and $N_{\mathrm{tst}}$ is the number of test data point.

**Figure 4:**
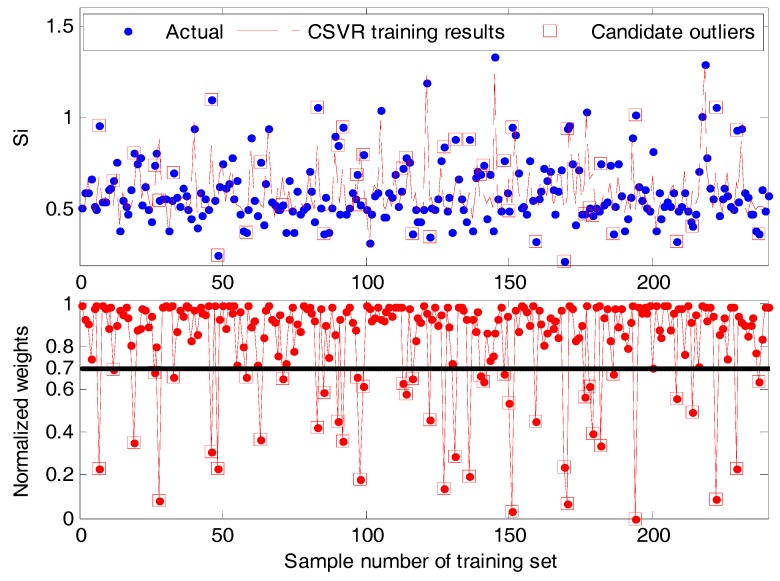
The trained CSVR model for fitting data with the normalized weights $\rho \left(e_{i}\right)$. As an affiliated product, those data with $\rho \left(e_{i}\right)<\bar{\rho }=0.7$ can be simply identified as candidate outliers.

The effect of a CSVR model is first investigated. After training, the main results, including the weighted terms $\rho \left(e_{i}\right)$, of a CSVR model are shown in Figure 4. Using the correntropy-based strategy, the outliers can be assigned with smaller weights different from most normal samples. As a result, the bad influence of outliers can be reduced. Here, the cut-off parameter is selected as $\bar{\rho }=0.7$. As shown in the bottom subplot of Figure 4, some candidate outliers can be identified directly. Finally, altogether 44 candidate outliers are chosen from all 240 training data. About 20% (44/240 = 18.3%) abnormal data, this indicates that the training data are noisy and contain several inconspicuous outliers. If the negative effect of these outliers are not removed, the prediction performance of established soft sensors cannot be good.

For comparison, the performance indices of the CSVR and LSSVR methods for the training data are listed in Table 1. The fitting results of both CSVR and LSSVR methods are not good. One main reason is that the data are noisy, non-Gaussian, and unevenly distributed. If the model fits all noisy training data, especially for the outliers, the over-fitting problem occurs. It can be noticed that the traditional LSSVR model cannot provide more information about the training data, treating all the training data equally. Different from LSSVR, the CSVR model can distinguish outliers from normal data and assign the training data with suitable weights.

**Figure 5:**
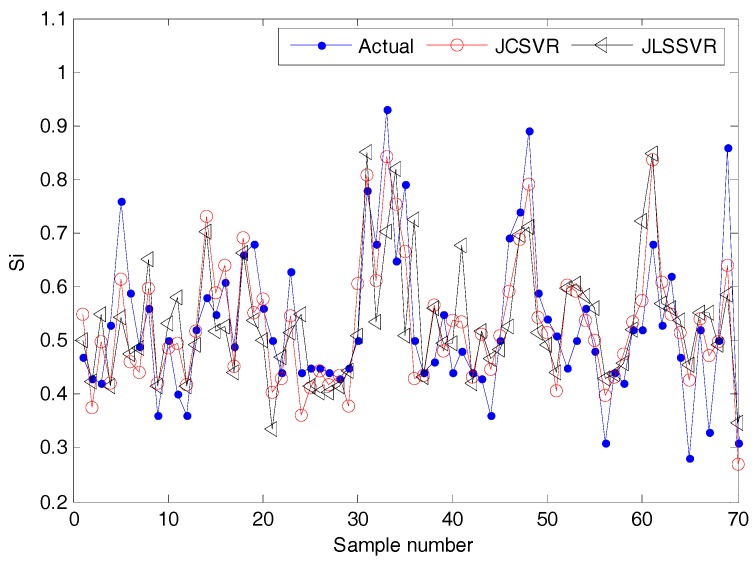
Comparison results of online silicon content prediction using JCSVR and JLSSVR models (test set).

**Figure 6:**
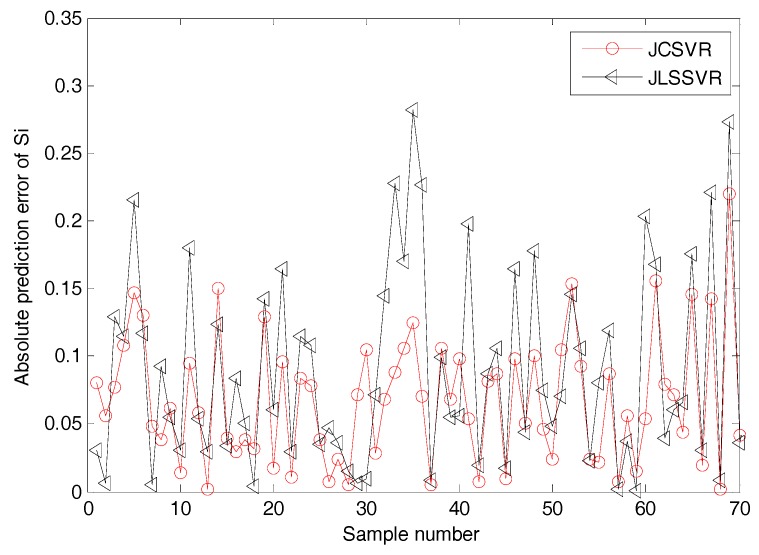
Comparison results of online silicon content prediction error using JCSVR and JLSSVR models (test set).

The dynamics may change in an industrial blast furnace. In such a situation, a fixed soft sensor model may be not accurate for future data [25,30]. Here, the proposed JCSVR-based method is compared with a recent local modeling method, named JLSSVR [25]. For the test data, the online prediction results and corresponding absolute prediction errors ($\left|y_{q}-{\hat{y}}_{q}\right|$) of JCSVR and JLSSVR methods are shown in Figure 5 and Figure 6, respectively. To show the result more clear, only the first 70 testing samples are plotted. As aforementioned, clean data are needed for online construction of a good local model. With some unwanted outliers, the prediction performance of a local model may be unreliable. Therefore, the prediction results shown in Figure 5 and Figure 6 indicate that JCSVR is superior to JLSSVR for industrial data-driven modeling problems with noisy data.

The main properties of the JCSVR, JLSSVR, CSVR, and LSSVR approaches are summarized in Table 2. Briefly, the outlier identification and local modeling are integrated into the JCSVR method. Detailed values about online silicon content prediction comparisons of four methods are listed in Table 3. It shows that the JCSVR method, for the test set, achieves the best prediction performance. Additionally, local models are generally more accurate than their global ones. For example, JCSVR shows better prediction performance than only using a CSVR model.

**Figure 7:**
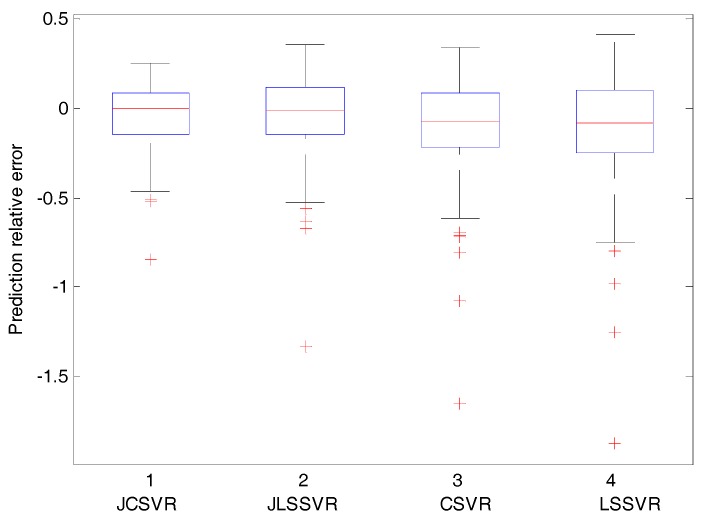
Comparison results of silicon content prediction relative error distribution with JCSVR, JLSSVR, CSVR, and LSSVR models (test set).

To show the relative prediction errors (i.e., $\frac{y_{q}-{\hat{y}}_{q}}{y_{q}}$) of four methods, their corresponding box plots are shown in Figure 7. The band inside the box is the median value, and the box edges denote the first and third quartiles. A few outliers are shown individually. Among four methods, JCSVR exhibits the narrowest distribution. The median value of JCSVR is nearest to 0. These results imply that JCSVR has the best prediction performance. One main reason is that the database is maintained continually. In contrast, without maintenance of the database, JLSSVR and LSSVR become unreliable and not suitable for long-term prediction. This is a common problem of traditional soft sensor models utilized in industrial processes. Based on all the prediction results and analysis, JCSVR is the most suitable one among all of the methods.

## 5. Conclusions

This work has proposed a correntropy-based local soft sensor modeling method for silicon content prediction when the collected data contain uncertainties. Its main distinguished characteristics are summarized. First, the soft sensor and outlier detection can be integrated into a CSVR modeling framework. By simply removing the candidate outliers, the updated historical data are more reliable for construction of local models. Second, by incorporating the database update into the clustering-based JCSVR method, better prediction performance can be achieved. Consequently, the proposed method can reduce the effect of outliers. Compared with several methods, better silicon content prediction results of JCSVR are obtained. There are still several interesting research directions worth investigating. First, other forms of correntropy can be adopted to adapt to the uncertainty of sensor data. Second, development of efficient feature extraction method for noisy data is interesting. Third, how to incorporate process knowledge to further improve the prediction accuracy is important and challenging.

## Figures and Tables

**Table 1:** Fitting results comparison of CSVR and LSSVR soft sensor models for the training set.

Soft Sensor Model	RMSE	RE (%)	HR (%)
CSVR [46]	0.116	21.49	73.33
LSSVR [24,47]	0.122	23.04	66.25

**Table 2:** Brief description of four different prediction models.

Prediction Model	Brief Description			
	Local and Unfixed	Outlier Detection	Main Pros	Main Cons
JCSVR	Yes	Yes	More suitable for noisy and uneven distributed data	Model selection is relatively difficult
JLSSVR [25]	Yes	No	Suitable for online modeling of nonlinear processes	Not robust for outliers
CSVR [46]	No	Yes	Suitable for construction of a global model with noisy data	Prediction accuracy for some local regions may not be enough
LSSVR [24,47]	No	No	A simple nonlinear modeling method	Not robust for outliers and relatively inaccurate for some local regions

**Table 3:** Comparison results of online silicon content prediction for the test set using four different models.

Prediction Model	RMSE	RE (%)	HR (%)
JCSVR	0.079	17.70	80.5
JLSSVR [25]	0.105	22.51	64.5
CSVR [46]	0.123	28.16	61.5
LSSVR [24,47]	0.141	31.29	52.5

## Abbreviations

The following abbreviations are used in this manuscript:

CSF	Cumulative Similarity Factor
CSVR	Correntropy Support Vector Regression
HR	Hit Rate
JCSVR	Just-in-time Correntropy Support Vector Regression
JLSSVR	Just-in-time Least Squares Support Vector Regression
LSSVR	Least Squares Support Vector Regression
RE	Relative root-mean-square Error
RMSE	Root Mean Square Error
SI	Similarity Index
SVR	Support Vector Regression
